# Costs of Inducible Defence along a Resource Gradient

**DOI:** 10.1371/journal.pone.0030467

**Published:** 2012-01-24

**Authors:** Christer Brönmark, Thomas Lakowitz, P. Anders Nilsson, Johan Ahlgren, Charlotte Lennartsdotter, Johan Hollander

**Affiliations:** Department of Biology, Aquatic Ecology, Lund University, Lund, Sweden; University of San Diego, United States of America

## Abstract

In addition to having constitutive defence traits, many organisms also respond to predation by phenotypic plasticity. In order for plasticity to be adaptive, induced defences should incur a benefit to the organism in, for example, decreased risk of predation. However, the production of defence traits may include costs in fitness components such as growth, time to reproduction, or fecundity. To test the hypothesis that the expression of phenotypic plasticity incurs costs, we performed a common garden experiment with a freshwater snail, *Radix balthica*, a species known to change morphology in the presence of molluscivorous fish. We measured a number of predator-induced morphological and behavioural defence traits in snails that we reared in the presence or absence of chemical cues from fish. Further, we quantified the costs of plasticity in fitness characters related to fecundity and growth. Since plastic responses may be inhibited under limited resource conditions, we reared snails in different densities and thereby levels of competition. Snails exposed to predator cues grew rounder and thicker shells, traits confirmed to be adaptive in environments with fish. Defence traits were consistently expressed independent of density, suggesting strong selection from predatory molluscivorous fish. However, the expression of defence traits resulted in reduced growth rate and fecundity, particularly with limited resources. Our results suggest full defence in predator related traits regardless of resource availability, and costs of defence consequently paid in traits related to fitness.

## Introduction

In recent years it has become increasingly recognised that, in addition to having constitutional defence adaptations, many organisms also respond to predation by modifying phenotypically plastic traits, such as behaviour, morphology or life-history strategies [e.g.], [ 1], [2,3]. The evolution of phenotypically plastic defence traits requires that prey experience a temporally or spatially variable predation pressure and that prey are able to have the correct phenotypic matching to the environment, i.e. they should have reliable cues of detecting the presence of a predator [2]. Moreover, the phenotypically plastic trait should have superior fitness in environments with predators compared to alternative phenotypes [4]–[7], i.e. for prey with predator induced defences the inducible defence should provide a benefit in terms of increased survival probability. However, predator mediated trait changes are expected to come with a fitness cost, since they are not expressed in the absence of predators. Although costs are predicted by theory it has been difficult to demonstrate the presence of costs in empirical studies [e.g., 2].

There are several types of costs potentially associated with phenotypic plasticity, including production costs to alter the phenotype compared to undefended phenotypes, maintenance costs, information acquisition costs, developmental instability costs and genetic costs [8]. In addition, there may also be underlying costs for plasticity *per se*, i.e. plastic genotypes are themselves costly irrespective of whether a trait is expressed or not [8]–[10]. In this paper we focus on costs associated with trait expression, and how the production costs influence trade-offs between traits that correlate with risk of predation. If the expression of phenotypically plastic traits is constrained by costs, then trait expression should depend on the environmental context, including differences in amount of resources available or the density of competitors [5], [11]–[15]. Various models predict that the optimal investment in defence changes along a gradient of resource availability/competitor density. For morphological defences where energy is allocated to build and maintain a defence structure at the cost of decreased growth or development rates, simple allocation models predict that investment in defence structures should be highest at high resource levels and/or at low densities of competitors [5]. At low resource/high competition levels the investment in defensive structures should be low, as all energy is needed to maintain basic life functions. Similarly, state-dependent models predict that behavioural responses to predation threat should be strongest at high resource levels/low competitor density, whereas at low resource levels prey have to be actively foraging in order to avoid starvation and thus show low levels of behavioural response to predator cues [14], [16]–[19].

Here, we investigate the expression of behavioural and morphological defence adaptations and how they co-vary along a gradient of intraspecific competition using the snail *Radix balthica* as a model organism. This is a phenotypically plastic species that has been shown to respond both behaviourally and morphologically to predation threat [20]–[22]. When it is exposed to chemical cues from molluscivorous fish it increases its refuge use and also develops a rounder shell shape. Roundness of the shell is considered to confer an increased resistance to predation by shell crushing predators as a rounder shape is correlated with increased crushing resistance, resulting in higher survival probability when encountered by molluscivorous fish [23]. Costs of expressing phenotypically plastic traits were quantified both as changes in behavioural and morphological defence as well as in traits related to fitness, such as growth and reproduction.

## Materials and Methods

### Experimental setup

The experimental setup consisted of eight 70 l opaque plastic tanks placed in a greenhouse. The tanks were aerated, filled with 10 mm of sand and two 20 cm PVC drainpipes that provided shelter for the fish. Half of the tanks were stocked with tench (*Tinca tinca*), a molluscivorous fish common in European ponds and lakes, and the remaining four tanks were fish-less controls. In each of the large tanks we placed five 2 l containers that were stocked with snails at the start of the experiment. The small tanks had a 10 cm opening, covered with plastic net (mesh 0.5 mm), in each short side, allowing for water exchange. A ceramic tile placed on the bottom and elevated 15 mm with legs provided a refuge for snails. To minimize fertilizing effects of fish on algal resources in the fish treatment tanks, plant fertilizer was added to maintain a total phosphorus level corresponding to eutrophic conditions (50 µg P l^−1^) in all tanks. Periphyton was allowed to colonize the containers 20 days prior to the start of the experiment and a constant light intensity was provided for all tanks and containers. Variation in the availability of food resources was governed by snail density only. Temperatures ranged between 19° C to 29° C in a light∶dark cycle of 16∶8 h.

### Study animals

Snails were collected in a fish-free pond 40 km southeast from Lund, southern Sweden. Approximately 100 adult *R. balthica* were allowed to reproduce in a 70 litre container and the egg capsules were removed and hatched in a 10 litre aerated aquarium. Juvenile snails measuring 1.6±0.2 mm were collected 14 days after hatching and placed haphazardly in the 2 l tanks. Snails were added to the small containers in a density gradient (2, 4, 8, 16 and 24 snails per container, respectively) such that all densities were represented within each large tank. Treatments were replicated four times.

### Predator

Tench were collected by electro-fishing in a pond 20 km north of Lund and acclimated to laboratory conditions for 11 days prior to the experiment. During this period they were fed fish food pellets. Initial length and biomass was 192.3±10.3 mm (mean ± SD) and 105.3±19.3 g, respectively. Two tench were placed in each predator treatment tank, and during the experiment they were fed 12 crushed *R. balthica* (length: 13.8±1.4 mm) per week.

The study complies with the current laws in Sweden; ethical concerns on care and use of experimental animals were followed under the permission approved for this study (M165-07) from the Malmö/Lund Ethical Committee.

### Behavioural assessment

During the entire experiment we monitored avoidance behaviours once a week. Individuals considered being in refuges, i.e. under the ceramic tile or above the water line [20], were counted. Snails in all other places were considered not showing any avoidance behaviour. The mean proportion of snails showing avoidance behaviour was calculated for each snail density.

### Shell measurements

The experiment was terminated after 12 weeks and the snails were deep frozen. At a later date, snails were thawed and soft tissues removed. Shells were scanned with the shell aperture down on a flatbed scanner (EPSON 2450 Photo). Images were analyzed using the image analyzing program SHAPE [24]. Since snails have few homologous points that can be used in landmark morphometrics, we chose to use elliptic Fourier analysis, as it captures the outline of the shell and thus the curved shape indicating shell roundness. The SHAPE program generates shape characteristics as principal components, and shape analysis is independent of size, position or rotation of the object. To interpret the loadings of the principal components, shape has to be visualized through inverting the Fourier transformation where after areas of shape variation can be identified. Retained principal components explained at least 5% of the total variation in shape.

Shell crushing resistance was estimated according to Osenberg and Mittelbach [25]. Each shell was put in a glass beaker and an empty beaker was placed on top and filled with sand until the shell was crushed. Beaker and sand was then weighed and the weight converted to crushing force (Newton) and size adjusted [26].

### Fitness traits

The final size of the snail shells were quantified as total length along the length axis of the shell, measured in the scanned images. Further, time at first reproduction was noted and the continued reproduction was monitored by removing egg capsules twice a week and counting eggs in a stereo microscope.

### Data analysis

Where required, data were transformed to meet the requirements for normality prior to analysis. Data were analyzed with a split-plot MANOVA design testing for predator treatment (fish or no fish), snail density with five levels, the interaction effect (treatment×density), and including aquaria identities nested within treatment as a blocking factor in the model [27]. This split-plot design was used to include the correct number of error degrees of freedom and to prevent the risk of pseudoreplication. Significant MANOVA effects were further analysed in univariate between-subject effect analyses for each response variable.

As we were interested in to what degree morphological traits are correlated with fitness traits and, further, if trait integration changes with environmental context (here intensity of competition), we contrasted the lowest density (n = 2 snails) with the highest density treatment (n = 24 snails), and calculated their respective phenotypic variance-covariance matrix [28], [29]. We standardized the covariances by dividing each pair of variables in correlations by their standard deviations to obtain a correlation matrix [27]. The covariance component estimates were calculated using the software H2boot [30] and the program produced a distribution from a bootstrap approach where we randomized the data 10 000 times in order to generate a null distribution which our results were tested against. The purpose was to investigate trait integrations and trade-offs (negative correlations), i.e. costs paid in fitness traits related to the development of various predation defence traits, at the extreme ends of densities used.

## Results

The results from the trait analysis can be divided into two categories. Traits that affect the probability of survival when encountered by a predator, including shell shape, shell crushing resistance and predator avoidance behaviour, and traits not directed to defending the organism but directly related to fitness, including final size, egg number and time to first reproduction. The multivariate analysis of variance (MANOVA) showed significant effects of fish cue treatment (Wilks λ = 0.046, *F*_7, 18_ = 53.744, *p*<0.001), snail density (Wilks λ = 0.015, *F*_28, 66.322_ = 5.228, *p* = <0.001), and the treatment×density interaction (Wilks λ = 0.060, *F*_28, 66.322_ = 2.795, *p*<0.001) on dependent variables. The factor aquaria nested within treatment was significant and indicates noise among the replicate units, which illustrates residual variation, possibly originating from discrepancies in recourse availability between aquaria. Variation in the residual potentially lowers the power of the model, making the interpretations of significant effects more robust. Results from the separate univariate ANOVAs are reported in table 1.

**Table 1:** Results showing treatment (control, predator), density (2, 4, 8, 16, 24 snails) and the interaction term treatment×density.

		Treatment			Density			Treatment	×Density	
Source	df	*F*	*P*	df	*F*	*p*	df	*F*	*p*	error df
										
PC 1	1	81.742	**<0.001**	4	0.818	0.526	4	0.658	0.627	24
PC 2	1	3.234	0.085	4	63.078	**<0.001**	4	14.629	**<0.001**	24
Crushing resistance	1	70.922	**<0.001**	4	1.831	0.156	4	0.147	0.962	24
Avoidance behaviour	1	48.455	**<0.001**	4	0.416	0.795	4	0.246	0.909	24
Final size	1	123.476	**<0.001**	4	84.076	**<0.001**	4	15.522	**<0.001**	24
Individual fecundity	1	49.383	**0.001**	4	38.204	**<0.001**	4	23.934	**<0.001**	24
Time to first reproduction	1	19.642	**<0.001**	4	1.431	0.254	4	1.067	0.395	24

Traits above the dotted line are defensive traits and traits below are more fitness related traits. Bold figures indicate significant effects.

### Defence traits

**Figure 1:**
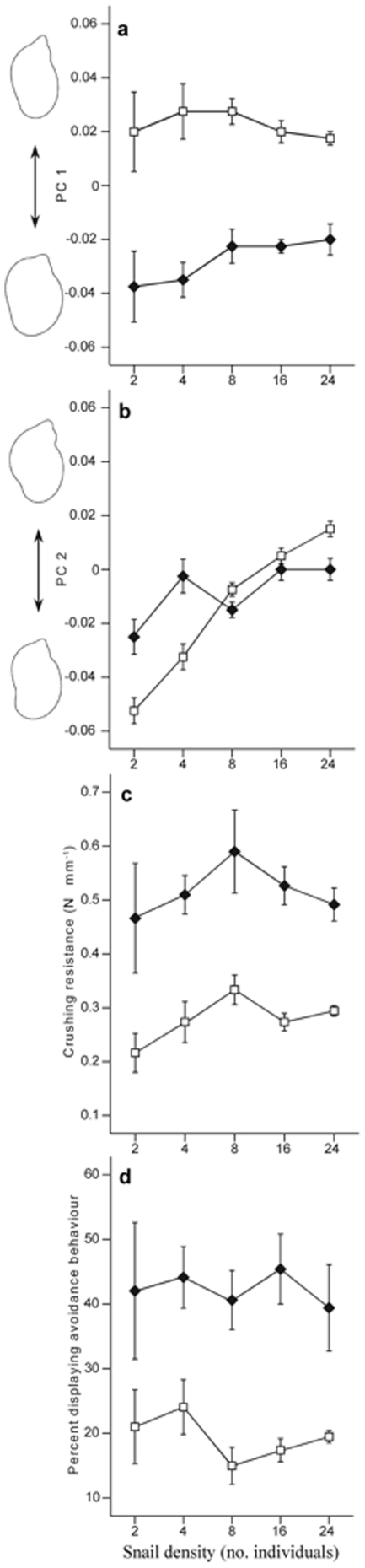
Showing defensive traits at increasing snail densities (2, 4, 8, 16 and 24 snails) in treatments with no fish (open squares) and in the presence of fish (closed diamonds). The effects of shell shape in PC 1 (a) and PC 2 (b). The visualized outline shell shape is on the left side. Positive scores in PC 1 have an outline with a narrow aperture and a long apex and negative scores generates an outline with a wider body whorl and a larger shell opening, as well as a lower apex. For PC 2, a widened second whorl and a narrower aperture generate positive scores, while a narrowing of this area and a widening of the aperture generate negative scores. Size corrected shell crushing resistance (c) and the proportion of snails showing predator avoidance behaviour (d). Values are mean and SE.

The cumulative contribution of the first two principal components in shell shape explained 79.2% of the variation. Positive scores on the first principal component (PC 1), which represented 50.8% of the variation in shape, were associated with a narrower aperture and a well defined, relatively long apex, whereas negative scores were associated with a wider body whorl and a larger shell opening, as well as a decrease in relative height of the apex. The shape of the snails in the control was characterized by positive scores, whereas snails from the predator treatment had more negative scores resulting in a significant predation effect. There was no effect of density on snail shape as characterised by PC 1, and no interaction effect (Fig. 1A, Table 1). The second principal component (PC 2) represented 28.4% of the variation and was primarily associated with a widening of the second last whorl for positive scores, and a narrowing of the second last whorl for negative scores. PC 2 showed no predator effect, but a significant density effect and an interaction (Fig. 1B, Table 1). Shell crushing resistance increased in the predator treatment, while there were no significant snail density or interaction effects (Fig. 1C, Table 1). The proportion of snails showing predator avoidance behaviour, i.e. crawl-out behaviour or hiding under refuge, was higher in the predator treatment but was consistent across densities resulting in no significant density or interaction effects (Fig. 1D, Table 1).

### Fitness traits

**Figure 2:**
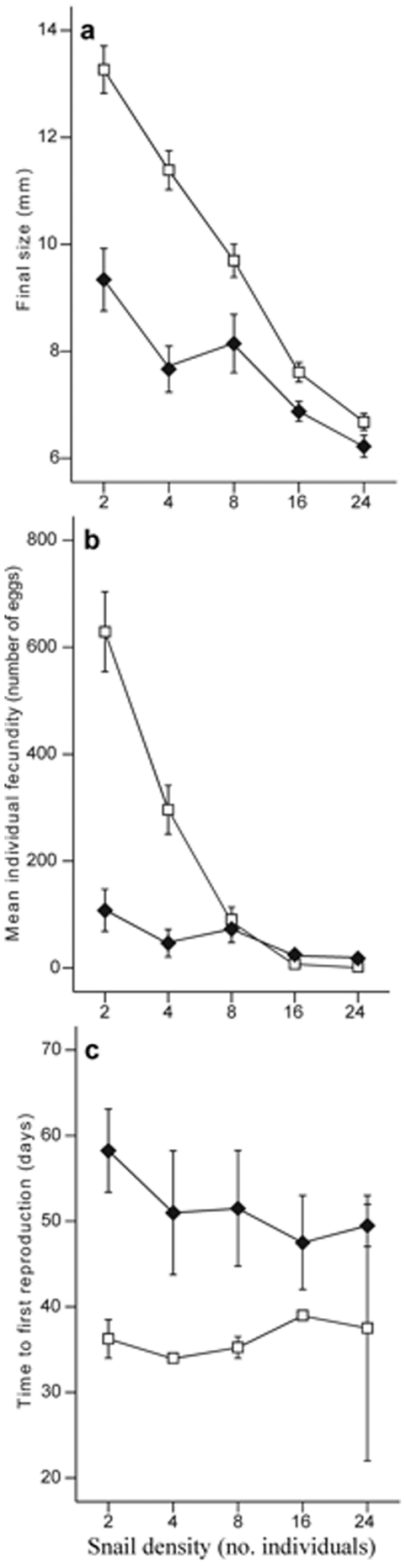
Showing fitness related traits at increasing snail densities (2, 4, 8, 16 and 24 snails) in treatments with no fish (open squares) and in the presence of fish (closed diamonds). The dependent variables are: final size of the snails (a), averaged individual fecundity (b), time in days to first reproduction (c). Values are mean and SE.

The predator treatment induced a cost in snail growth, shown as a smaller final size in the predator compared to the control treatment (Fig. 2A, Table 1). There was also a significant cost associated with snail density, with reduced growth and final size with increasing snail densities. The difference between predator treatment and control was large at low densities, but gradually diminished at higher densities, resulting in a significant predator×density interaction effect.

The costs induced by predator cues also showed in individual fecundity, i.e. the average number of eggs produced per capita, that was significantly lower in the presence of predator cues. There was also a significant effect of snail density and a predator×density effect on snail fecundity (Fig. 2B, Table 1). Snails in the predator treatment suffered a cost from a longer time to reproduction, while this cost was not affected by snail density or the interaction term (Fig. 2C, Table 1).

### Trait correlations

**Figure 3:**
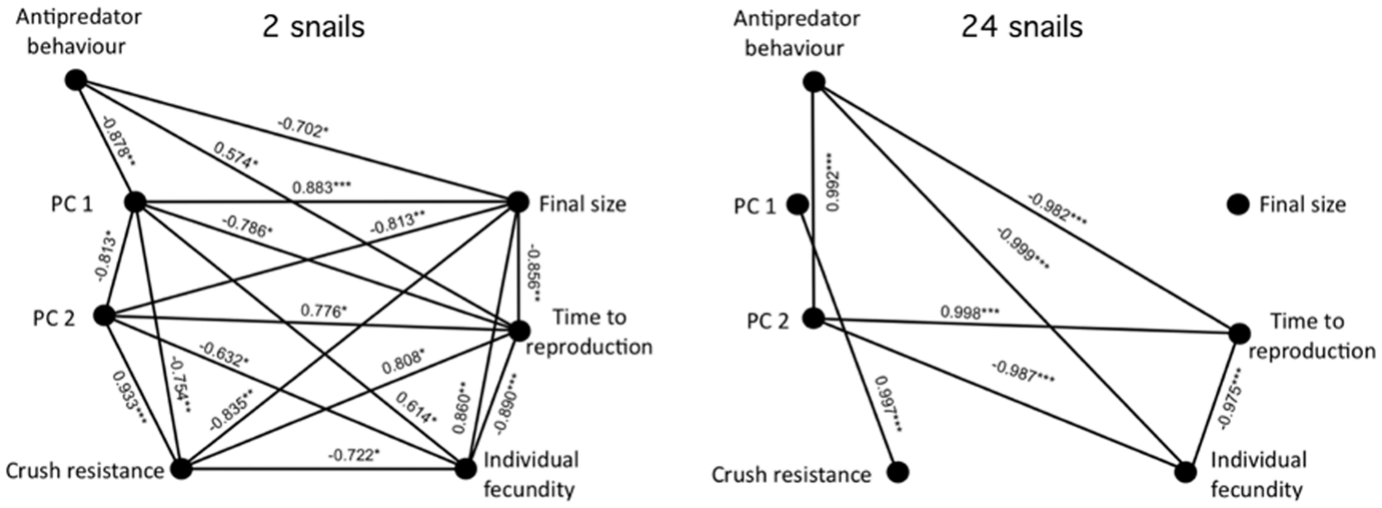
Trait correlations for low and high densities. The low density including 2 snails (a), while the high density encompassing 24 snails (b). Lines designate a significant correlations between traits, and stars indicate P-values: *<0.05; **<0.01; ***<0.001.

The correlation matrix had 21 possible correlations between traits, and at low snail density 18 correlations were significant (Fig. 3A). Snails with a rounder shell in PC1 also had a wider body whorl in PC2 and a smaller final size. Snails corresponding with such a shell shape did also spend more time in the refuge. The shape in PC 2 was associated with a high crushing resistance. Both PC2 and crush resistance were negatively correlated with the fitness components final size and individual fecundity, and were associated with increased time to reproduction. A high antipredator behaviour was correlated with costs in final size and time to reproduction. At high snail density (n = 24) numerous significant correlations disappeared (Fig. 3B), but PC2 remained an important contributor to costs in fecundity and time to reproduction, and antipredator behaviour also affected these fitness-related traits, by resulting in snails producing less eggs and reproducing earlier.

## Discussion

We found evidence of *R. balthica* paying costs in fitness-related growth and reproductive traits when expressing morphological and behavioural defences against predation. Predator cues induced a strong response in the expression of morphological and behavioural defence traits in *R. balthica*. Theory predicts that trait expression should decrease with decreasing resource levels if there is a cost associated with the inducible defence trait. Here, we showed that defence traits were consistently expressed independent of the degree of intraspecific competition and, thus, *R. balthica* invest in defence independently of resource levels in the environment. We assume that resource availability is directly related to snail density, an assumption that is strengthened by earlier studies on competition in snails where increasing snail density resulted in reduced biomass of periphytic algae [31], [32]. However, in some species, the effect of decreasing resource availability density on the expression of inducible defences may not be directly comparative to the effects of increasing competitor densities [33], for example when individuals defend a supply of food against competitors. However, we have not observed such interference competition in *R. balthica*. The investment in defence traits independently of resource levels suggests that the expression of defence traits is highly prioritized in this species and that predation is a strong selection pressure. The costs of expressing defences were instead found in a number of fitness traits associated with growth and reproduction. Most fitness traits may have evolved in response to a number of environmental factors and would thus show larger inconsistency in relation to predation risk and conspecific competition. In contrast, defensive traits often evolve under independent directional selection to a specific predator [34] and should for that reason demonstrate less variability in their phenotypic response and express their maximal phenotype across the complete resource gradient [35]. As a result, the variability in fitness traits is illustrated in the interaction term, while the predator specific response would lack this interaction and respond to predators only, as we observed in most defence traits (Table 1).

### Morphological traits

The most obvious defensive structure in a snail is the shell. In our experiment we analyzed aspects of shell shape and the effect on shell crushing resistance. Exposure to predator cues resulted in a rounder shell shape, with a wider body whorl and a larger shell opening. These changes in shell morphology resulted in a shell that was more resistant to shell crushing forces, i.e. a change that should be highly adaptive when exposed to predation from shell-crushing predators such as fish. In a laboratory experiment DeWitt and co-workers [23] found that snails with an induced, more round morphology were rejected to a higher degree when encountered by molluscivorous sunfish.

If building a thicker shell with a more round shape is more costly than then the phenotype in absence of predators, then this cost may be traded off in energy that could have been used for expression of other traits or for growth and reproduction. However, the expression of costs may be dependent on resource levels or, as in this case, competitor density. However, we found no effect of density and no significant interaction between predator presence and density interaction in the first principal component, which explained most of the variation in shell shape. The second principal component, which explained a minor part of variability in shell shape, was affected by a significant interaction effect; the effect of snail density was much less pronounced in predator treatments than in controls. Our results are in contrast to allocation models that predict that if there are costs associated with the defence adaptation then the investment in the defence structure should be highest at high resource levels/low competition intensity [5]. Several studies have investigated the expression of morphological, phenotypically plastic defences in prey organisms along resource or competition gradients, but results have not been consistent. Hoverman et al. [36] studied another freshwater snail species, *Helisoma trivolvis* that show predator induced changes in shell morphology, but found no interaction between resource levels and predator treatment on the expression of shell shape. Some studies on tadpoles have found predator×density interactions that follow predictions from allocation models, i.e. reduced investment in defence structures at high competition [9], [37], [38], whereas others have found no significant interaction effect [14], [35], [39]. Clearly, further studies are needed in this area.

### Behavioural traits

Snails spent more time in the refuge or out of water in the predator treatment. Such behavioural defence responses to predation threat have been commonly observed in freshwater snails [20], [36], [40]–[43]. Increased refuge use and lowered activity decreases the time available for foraging, resulting in reduced growth and reproduction [1], [15], [44]. Predictions suggest that such costs should be less important at high resource levels or low competition intensity [12], [15], [45]. However, the snails in our experiment showed a response to fish predation threat that was independent of snail density, which is not consistent with state-dependent models. Turner [45] demonstrated a density-dependent risk response in *Helisoma trivolvis* snails, where increased nutrient resources caused snails to minimize refuge use. Conversely, Hoverman et al. [36] found no effect of resource level on defence behaviours, and similar results have also been reported in tadpoles [14], [35], [37], [39]. The lack of an interaction effect in our study suggest that predation risk dominates over resource availability and that individual *R. balthica* snails always show their maximal avoidance behaviour in response to predation threat, independent of costs.

### Fitness traits

There was a strongly negative effect of predator cues on a number of fitness traits associated with growth and reproduction, indicating a cost of expressing defensive traits. The relative effect of predation threat on final size was largest at low snail densities. Final size declined sharply with increased density and the effect was more obvious in the predator free treatment resulting in a considerably smaller predator effect at higher densities. This may be a result of low resource levels at high densities that leaves less to be consumed by competing snails, regardless if they spend time foraging or not. Strong intraspecific and/or interspecific competition has also been observed in other freshwater snails [31], [46], [47]. The cost of reduced growth because of decreased feeding time, when resources are scarce, may therefore not necessarily be higher in the risky environment. Alternatively, prey cannot risk starvation by reducing feeding in the predator environment [12], [15]. However, at abundant resources, the lower rates are suggested to emerge because of a suppressed behavioural activity determining foraging rates [48]. Our results are consistent with several studies and theoretical predictions that correlate predation threat with resource levels, and which have shown an overall positive relationship on growth at lower prey densities. However, this effect weakens and almost fades out at higher prey densities [12], [15], [37], [45], [48].

Reproductive rate in *R. balthica* was reduced in the presence of predators and there was also a strong negative effect of intraspecific competition. The effect of competition was especially pronounced in the control treatment, whereas the response to density in the predator treatment was relatively lower, and at high densities the egg production in the predator treatment even exceeded the control group. Furthermore, the onset of reproduction was later in the predator treatment, but there was no effect of density. This effect of predator presence inducing a delayed time of first reproduction in snails has been observed before [36]. Crowl and Covich [49] found that the freshwater snail *Physella virgata* exhibits a more rapid growth rate in the presence of a predator and reproduces at a later age.

### Trait correlations

Previous studies that have examined the costs and benefits of alternative phenotypes in a suite of traits, as well as different types of traits, have found relatively few trait correlations among morphological defence traits and fitness component traits [34]. In contrast, our experiment with *R. balthica* showed eighteen trait correlations among traits in the low density treatment and seven correlations in the high density treatment. Among the morphological defence traits, shell shape and crush resistance against mechanical force appear to be vital against molluscivorous fish [23]. But while the morphological defence trait includes fitness benefits, traits related to fitness components, such as reduced growth and fecundity, demonstrate costs. Moreover, van Kleunen and Fisher [50] discuss the rate of plasticity, which may be reduced by resource limitations, and as demonstrated in this study, traits related to fitness are reduced when the density of snails is high. Thus, the reaction norms are lowered to a minimum, as phenotypic plasticity is masked due to indirect reduction as a result of competition. Also, correlations among defence traits and fitness traits follow the same trend; the number of correlations was much lower in the high density treatment, even if all correlations in the high density treatment except one corresponded with correlations detected in the low density treatment. The correlations observed in the high density treatment were certainly strong and may indicate less developmental noise in a harsh environment with predation threat and amplified competition.

In conclusion, we have shown a dynamic response to a predator-induced defence among multiple characters and how plastic defensive traits affect fitness values over a resource gradient. Our results, in part, support previous studies and conceptual models, while other findings show a contradiction to similar investigations. We found that predator exposed *Radix balthica* express a consistent investment in morphological and behavioural defence traits independent of intraspecific competition. Our results suggest that the snails are allocating phenotypic production costs to related traits not associated with survival, but to characters linked to life history traits. Furthermore, the trait integration of correlated responses was low and may imply that the few correlations detected in this study are predator specific, as previously shown among several species of larval anurans [34]. Trait-specific costs are challenging to identify, particularly to reveal how traits are allocated within species and how species invest in inducible defences, especially under more realistic ecological conditions. The evolution of phenotypic plasticity obviously deserves further empirical consideration in future work.
